# MicroRNA Signatures in Tumor Tissue Related to Angiogenesis in Non-Small Cell Lung Cancer

**DOI:** 10.1371/journal.pone.0029671

**Published:** 2012-01-25

**Authors:** Tom Donnem, Christopher G. Fenton, Kenneth Lonvik, Thomas Berg, Katrine Eklo, Sigve Andersen, Helge Stenvold, Khalid Al-Shibli, Samer Al-Saad, Roy M. Bremnes, Lill-Tove Busund

**Affiliations:** 1 Department of Oncology, University Hospital of North Norway, Tromsφ, Norway; 2 Institute of Clinical Medicine, University of Tromsφ, Tromsφ, Norway; 3 Department of Clinical Pathology, University Hospital of North Norway, Tromsφ, Norway; 4 Institute of Medical Biology, University of Tromsφ, Tromsφ, Norway; 5 Department of Pathology, Nordland Central Hospital, Bodφ, Norway; Queen Elizabeth Hospital, Hong Kong

## Abstract

**Background:**

Angiogenesis is regarded as a hallmark in cancer development, and anti-angiogenic treatment is presently used in non-small cell lung cancer (NSCLC) patients. MicroRNAs (miRs) are small non-coding, endogenous, single stranded RNAs that regulate gene expression. In this study we aimed to identify significantly altered miRs related to angiogenesis in NSCLC.

**Methods:**

From a large cohort of 335 NSCLC patients, paraffin-embedded samples from 10 patients with a short disease specific survival (DSS), 10 with a long DSS and 10 normal controls were analyzed. The miRs were quantified by microarray hybridization and selected miRs were validated by real-time qPCR. The impacts of different pathways, including angiogenesis, were evaluated by Gene Set Enrichment Analysis (GSEA) derived from Protein ANalysis THrough Evolutionary Relationship (PANTHER). One of the most interesting candidate markers, miR-155, was validated by *in situ* hybridization (ISH) in the total cohort (n = 335) and correlation analyses with several well-known angiogenic markers were done.

**Results:**

128 miRs were significantly up- or down-regulated; normal versus long DSS (n = 68) and/or normal versus short DSS (n = 63) and/or long versus short DSS (n = 37). The pathway analysis indicates angiogenesis-related miRs to be involved in NSCLC. There were strong significant correlations between the array hybridization and qPCR validation data. The significantly altered angiogenesis-related miRs of high interest were miR-21, miR-106a, miR-126, miR-155, miR-182, miR-210 and miR-424. miR-155 correlated significantly with fibroblast growth factor 2 (FGF2) in the total cohort (r = 0.17, P = 0.002), though most prominent in the subgroup with nodal metastasis (r = 0.34, P<0.001).

**Conclusions:**

Several angiogenesis-related miRs are significantly altered in NSCLC. Further studies to understand their biological functions and explore their clinical relevance are warranted.

## Introduction

Lung cancer is the number one cause of cancer related mortality in both men and women, and approximately 80% are non-small cell lung cancer (NSCLC) [1]. Despite emerging improvements in early diagnosis and treatment modalities, the overall 5-year survival is at meager 15%. TNM (Tumor, Nodes, Metastasis) stage has been regarded the most important prognostic variable as early stage disease is a prerequisite for complete surgical resection necessary for potential cure. Treatment responses and side effects from new treatment options have been closely related to different histological entities of NSCLC [2]–[4]. However, targeted therapies directed against specific cellular alterations require a precise sub-classification of NSCLC which is largely beyond today's staging and routine diagnostic histopathological techniques [5].

MicroRNAs (miRs) are small non-coding, endogenous, single stranded RNAs that regulate gene expression [6]–[9]. By regulating the gene expression at a posttranscriptional level, miRs have a large impact on a wide variety of pathways, including different pathways related to cancer development. Several studies have explored the deregulation of different miRs in NSCLC and their potential oncogenic and tumor suppressor functions as well as their prognostic impact [10]–[20].

Some miRs appear to be involved in angiogenesis [21]–[24]. Angiogenesis is a process of new blood vessel formation from pre-existing ones, and plays a key role in tumor development [25]. Angiogenic inhibitors are used in NSCLC treatment. The monoclonal antibody bevacizumab in combination with chemotherapy is FDA approved as a treatment option in metastatic non-squamous NSCLC patients, and many tyrosine kinase inhibitors targeting angiogenic pathways are promising [3], [26].

The first evidence showing miRs in the regulation of angiogenesis came from Dicer knockout mice. Dicer is a critical enzyme in miR synthesis. The Dicer-deficit mice died early during development due to the thinning of vascular walls and severe disorganization of the network of blood vessels [27]. Besides, another murine study showed that a subset of miRs altered during the angiogenic switch became oppositely regulated in response to anti-angiogenic treatment [23].

In a large unselected NSCLC cohort we have previously studied the prognostic impact of several key angiogenic growth factors and receptors as well as the prognostic role of miR-155 and miR-126 by *in situ* hybridization [28]–[38]. Herein, we evaluated the miR expression profiles in NSCLC by using tissue from selected patients from this cohort. We aimed to identify interesting angiogenesis-related miR candidates for further potential large-scale and in-depth studies.

## Results

### Patients

Demographic, clinical, and histopathological variables in the patient groups (long DSS, short DSS and controls) are shown in Table 1. The median DSS were 8.0 (range 6.5–9.9) months and 160.5 (range 60.5–184.3) months in the low and high DSS group, respectively. There were four adenocarcinomas and six squamous cell carcinomas in each group.

**Table 1:** Characteristics of 20 non-small cell lung cancer (NSCLC) patients and ten normal controls; NSCLC_01-10 with a short disease-specific survival (DSS), NSCLC_11-20 with a long DSS and 10 normal controls (Norm_01-10).

Patient ID	Histology	DSS (months)	Status	Stage	T	N	M	Differentiation	Age	Sex
NSCLC_01	AC	6.5	LC Dead	IIa	2a	1	0	Moderate	53	M
NSCLC_02	AC	7.0	LC Dead	IIa	2a	1	0	Poor	73	M
NSCLC_03	SCC	7.3	LC Dead	IIIa	1b	2	0	Poor	62	M
NSCLC_04	AC	7.4	LC Dead	IIa	1b	1	0	Moderate	74	M
NSCLC_05	AC	7.6	LC Dead	IIb	3	0	0	Poor	54	M
NSCLC_06	SCC	8.4	LC Dead	IIb	3	0	0	Well	53	M
NSCLC_07	SCC	8.4	LC Dead	IIb	3	0	0	Poor	74	M
NSCLC_08	SCC	9.3	LC Dead	IIa	2a	1	0	Poor	74	M
NSCLC_09	SCC	9.6	LC Dead	IIb	3	0	0	Poor	68	F
NSCLC_10	SCC	9.9	LC Dead	IIb	3	0	0	Poor	70	F
NSCLC_11	SCC	147.8	Alive	IIa	2a	1	0	Moderate	53	F
NSCLC_12	AC	74.1	Alive	IIa	2b	0	0	Moderate	75	M
NSCLC_13	AC	60.5	Alive	Ia	1b	0	0	Moderate	75	F
NSCLC_14	SCC	147.8	Alive	IIa	2b	0	0	Moderate	60	M
NSCLC_15	SCC	163.8	Alive	IIIa	3	1	0	Poor	68	M
NSCLC_16	AC	170.5	Alive	IIa	2b	0	0	Moderate	47	M
NSCLC_17	SCC	158.7	Non-LC Dead	Ib	2a	0	0	Moderate	69	M
NSCLC_18	AC	177.4	Alive	IIb	3	0	0	Moderate	64	M
NSCLC_19	SCC	184.3	Non-LC Dead	Ia	1b	0	0	Moderate	61	M
NSCLC_20	SCC	162.3	Alive	Ia	1b	0	0	Moderate	53	M
*NORM_01	AC	6.5	LC Dead	IIa	2a	1	0	Moderate	53	M
*NORM_02	AC	7.0	LC Dead	IIa	2a	1	0	Poor	73	M
*NORM_03	SCC	8.4	LC Dead	IIb	3	0	0	Well	53	M
*NORM_04	SCC	147.8	Alive	IIa	2a	1	0	Moderate	53	F
*NORM_05	AC	74.1	Alive	IIa	2b	0	0	Moderate	75	M
*NORM_06	AC	60.5	Alive	Ia	1b	0	0	Moderate	75	F
*NORM_07	SCC	163.8	Alive	IIIa	3	1	0	Poor	68	M
*NORM_08	AC	170.5	Alive	IIa	2b	0	0	Moderate	47	M
*NORM_09	AC	177.4	Alive	IIb	3	0	0	Moderate	64	M
*NORM_10	SCC	184.3	Non-LC Dead	Ia	1b	0	0	Moderate	61	M

Abbreviations: SCC, squamous cell carcinoma; AC, adenocarcinoma; DSS; LC, lung cancer related; T, tumor; N, nodal status; M, metastasis.

*Samples from normal lung tissue sites in ten NSCLC patients (NSCLC_01, NSCLC_02, NSCLC_06, NSCLC_11, NSCLC_12, NSCLC_13, NSCLC_15, NSCLC_16, NSCLC_18 and NSCLC_19) were included in the study (NORM_01 - NORM_10).

### Micro RNA expression analysis

**Figure 1:**
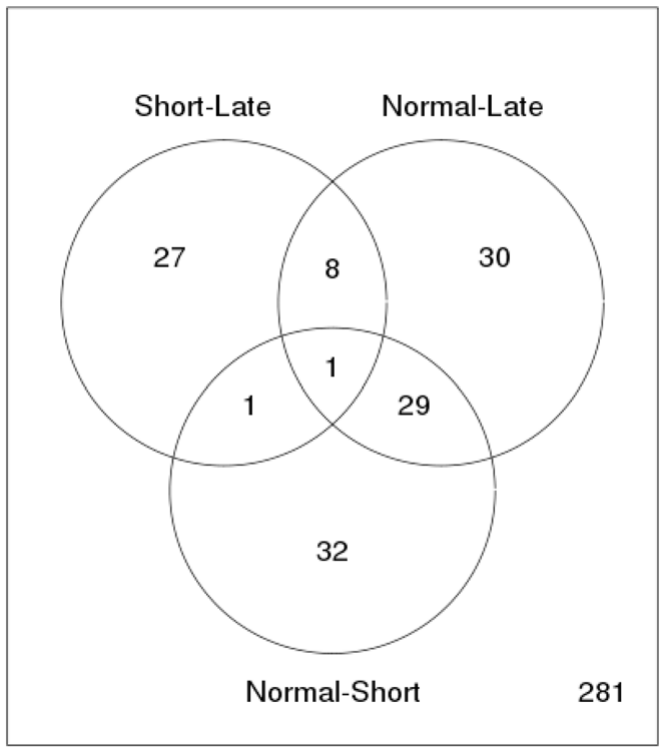
Comparison of survival groups of NSCLC patients. The Venn diagram shows the number of all differentially expressed miRNAs across different comparisons: Tissue from NSCLC patients with short survival versus tissue from NSCLC patients with long survival, tissue from normal lung versus tissue from NSCLC patients with long survival and tissue from normal lung versus tissue from NSCLC patients with short survival. Out of 281 miRs evaluated, the number of differential expressed miRs with FDR adjusted P<0.1 (total n = 128) of each comparison is indicated.

**Figure 2:**
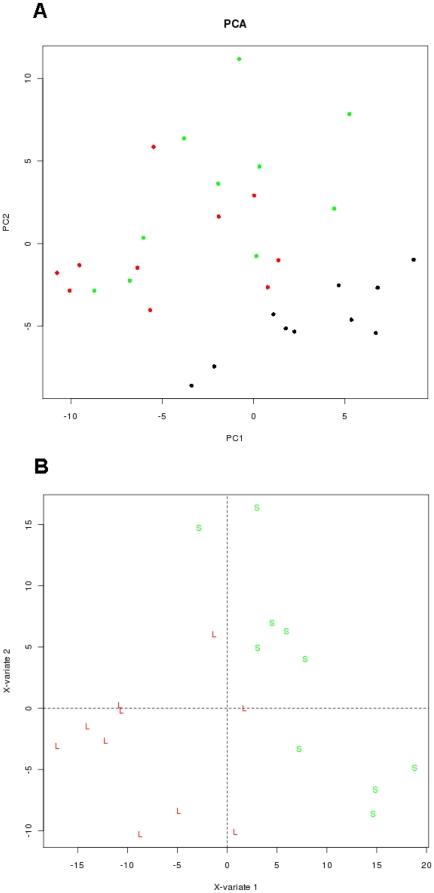
Principal component analysis (PCA) and partial least square analysis (PLS) on different NSCLC patient samples. (A) Two-dimensional PCA of miRs, derived from 20 patients with NSCLC and 10 tissue samples from normal lung tissue, showing separation of the two sample groups. (B) The plot depicts components 1 and 2 of PLS model which used survival time as a scoring criteria. The analysis clearly separates the tissue sample groups for short and long survival NSCLC patients. All samples are colour coded according to group: Black: Normal patient samples; green: Samples from patients with short survival; red: Samples from patients with long survival.

Out of 281 miRs, Figure 1 shows the number and distribution of 128 differentially expressed miRs significant across different comparisons in the study (P<0.1 adjusted for false discovery rate, FDR). The respective -up and down-regulated (−1,1) miRs are presented in Table S1. miR-182 was the only miR altered in all three combinations; normal versus short DSS, normal versus long DSS and short versus long DSS. A Principal component analysis (PCA) (Figure 2A) on the entire sample set of expression values revealed that the main principle component of variation separates normal samples from NSCLC samples. To highlight the difference between the groups of NSCLC tumor samples a bridge partial least squares (PLS) model was used to extract a subspace where the group differences are more obvious than in PCA (Figure 2B). Table 2 shows the ten miRs with highest fold change stratified into the groups long survival versus normal control and short survival versus normal control. Table 3 shows the miRs with highest fold change in short versus long survival groups.

**Table 2:** Ten each of the differentially expressed miRs that are up- or down-regulated the most ranged by fold change (long survival versus normal control and short survival versus normal control).

	Long survival versus normal			Short survival versus normal		
	miR	Fold change	P-adjusted*	miR	Fold change	P-adjusted*
**Up-**	miR-205	8.75	0.0273	miR-1308	3.92	0.0117
**regulated**	miR-21	3.68	0.0090	miR-21	3.63	0.0094
	miR-1308	2.89	0.0532	miR-182	3.32	0.0008
	miR-93	2.11	0.0443	miR-31	2.87	0.0362
	miR-1274a	1.99	0.0675	miR-205	2.75	0.2917
	miR-182	1.80	0.0939	miR-193b	2.31	0.0062
	miR-708	1.75	0.0039	miR-1259	2.28	0.0008
	miR-210	1.74	0.0070	miR-93	2.23	0.0337
	miR-1259	1.64	0.0427	miR-106a	2.23	0.0933
	miR-106b	1.62	0.1644	miR-183	2.20	0.0304
**Down-regulated rrregulatedregulated**	miR-451	5.74	0.0427	miR-451	5.85	0.0397
**regulated**	miR-126	4.53	0.0005	miR-126	4.09	0.0008
	miR-30a	3.14	0.00006	miR-30a	2.81	0.0003
	miR-30b	3.07	0.0124	miR-140-3p	2.68	0.0035
	miR-30c	2.45	0.0035	miR-143	2.33	0.0787
	miR-140-3p	2.45	0.0070	miR-126*	2.28	0.0035
	miR-126*	2.41	0.0016	miR-145	2.22	0.0070
	miR-145	2.28	0.0061	miR-30b	2.07	0.0958
	let-7a	2.25	0.0480	miR-29c	2.01	0.0934
	miR-125a-5p	2.19	0.00001	miR-30d	1.89	0.0200

*P-adjusted; corrected for false discovery rate (FDR).

**Table 3:** Ten each of the differentially expressed miRs that are up- or down-regulated the most ranged by fold change (short survival versus long survival).

	Short survival versus long survival		
	miR	Fold change	P-adjusted*
**Up-**	miR-31	2.00	0.161
**regulated**	miR-182	1.85	0.084
	miR-106a	1.73	0.245
	miR-183	1.72	0.094
	let-7a	1.68	0.194
	miR-151-5p	1.64	0.093
	miR-138-1*	1.61	0.073
	miR-98	1.56	0.089
	miR-424	1.53	0.044
	miR-193b	1.50	0.178
**Down-**	miR-205	3.16	0.232
**regulated**	miR-142-3p	1.96	0.084
	miR-557	1.65	0.084
	miR-720	1.52	0.078
	miR-378	1.44	0.070
	miR-708	1.41	0.075
	miR-29c	1.39	0.076
	miR-552	1.35	0.084
	miR-27a*	1.32	0.035
	miR-155	1.31	0.095

*P-adjusted; corrected for false discovery rate (FDR).

### Pathway analysis

**Figure 3:**
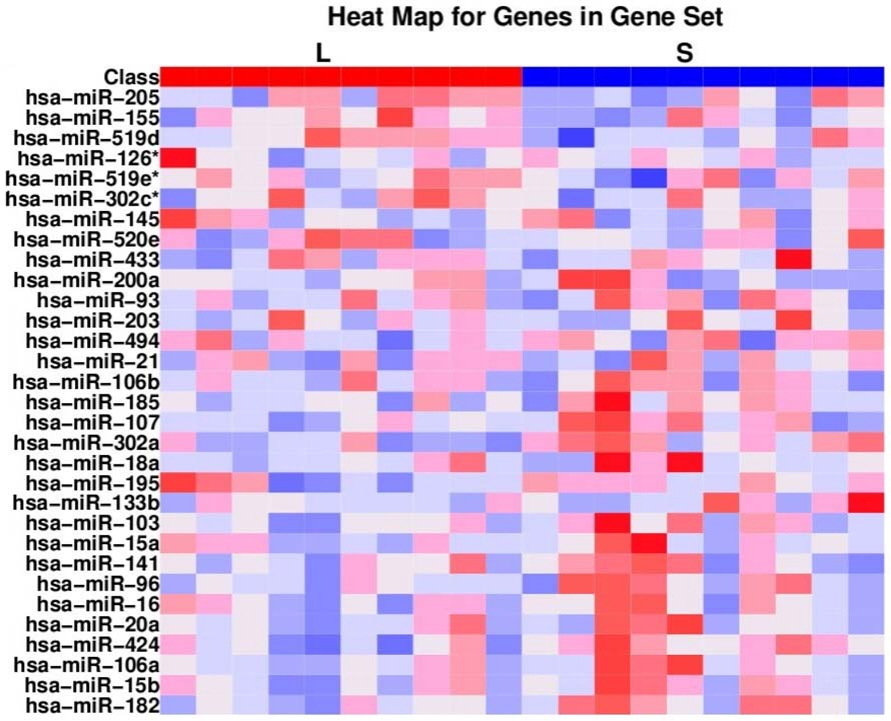
Heat map showing expression of 31 microRNAs (miRs) included in angiogenesis pathway gene set. The difference in miR expression between tumor samples from NSCLC patients with long (L) and short (S) survival is shown. miR expression values are shown in a spectrum where down-regulated is blue and up-regulated is red.

Table 4 shows results from the GSEA. The gene set connected to 31 angiogenesis-related miRs revealed the highest gene set nominal enrichment score (NES = −1.17, nominal p-value 0.28, FDR 0). Figure 3 shows the heat map representing 31miRs connected to the angiogenic gene set.

**Table 4:** Impacts of the different pathways evaluated by Gene Set Enrichment Analysis (GSEA) derived from Protein Analysis THrough Evolutionary Relationship (PANTHER).

GS	Size	Pathway	NES	NOM p-value	FDR
P00005	31	Angiogenesis	−1.17	0.28	0
P00027	17	Heterotrimeric G-protein signaling pathway-Gq alpha and Go alpha mediated pathway	−1.16	0.28	0
P00004	42	Alzheimer disease-presenilin pathway	−1.12	0.33	0.35
P00047	37	PDGF signaling pathway	−1.12	0.32	0.26
P00029	37	Huntington disease	−1.08	0.41	0.35
P00059	21	p53 pathway	−1.06	0.43	0.31
P00006	28	Apoptosis signaling pathway	−1.06	0.50	0.28
P00048	18	PI3 kinase pathway	−1.04	0.46	0.27
P00031	45	Inflammation mediated by chemokine and cytokine signaling pathway	−1.03	0.49	0.26
P00019	19	Endothelin signaling pathway	−1.00	0.50	0.33
P00036	35	Interleukin signaling pathway	−0.98	0.51	0.37
P00057	62	Wnt signaling pathway	−0.98	0.54	0.34
P00052	44	TGF-beta signaling pathway	−0.89	0.66	0.56
P00034	43	Integrin signalling pathway	−0.89	0.68	0.53
P00012	40	Cadherin signaling pathway	−0.87	0.66	0.55
P00046	22	Oxidative stress response	−0.86	0.67	0.55
P00026	33	Heterotrimeric G-protein signaling pathway-Gi alpha and Gs alpha mediated pathway	−0.85	0.68	0.57
P04398	28	p53 pathway feedback loops 2	−0.83	0.74	0.58
P00016	23	Cytoskeletal regulation by Rho GTPase	−0.81	0.71	0.59
P00060	44	Ubiquitin proteasome pathway	−0.73	0.81	0.81
P00007	16	Axon guidance mediated by semaphorins	−0.67	0.87	0.90

GS, gene set; Size, numbers of miRs included; NES, nominal enrichment score; NOM p-value, nominal p-value; FDR, false discovery rate.

### PCR validation

**Figure 4:**
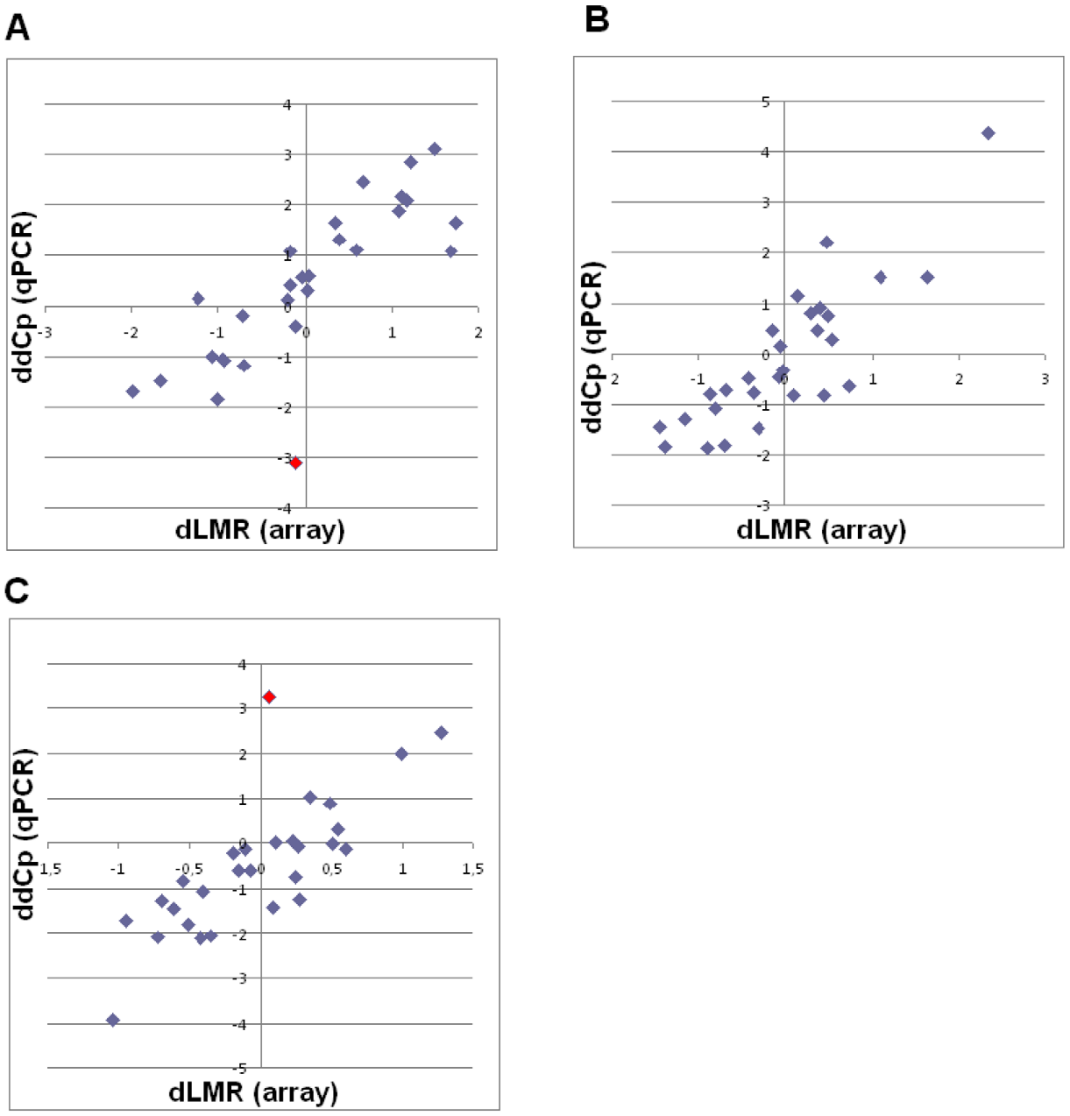
Scatter plot comparing microarray hybridization (all ten samples in each group) and qPCR data (five selected samples from each group: NSCLC_03, NSCLC_05, NSCLC_08, NSCLC_10, NSCLC_15, NSCLC_16, NSCLC_17, NSCLC_18, NSCLC_19, NSCLC_20, NORM_03, NORM_04, NORM_08, NORM_09 and NORM_10) and 28 miRs according to Table S2. Comparison between microarray hybridization (dLMR, difference in average expression levels between sample groups, log2 scale) and qPCR (ddCP, difference in average expression levels between sample groups, log2 scale) data, correlation coefficient = r (Pearson): (A) low versus normal r = 0.81, P<0.001; (B) high versus normal r = 0.85, P<0.001; (C) high versus low r = 0.85, P<0.001. In red; miR-150.

Scatter plots comparing microarray hybridization and qPCR data from 28 selected miRs are shown in Figure 4. The included miRs, and data from all three combinations (both hybridization and qPCR) are listed in Table S2. There were strong and significant correlation between the hybridization and qPCR data: Short versus normal r = 0.81, P<0.001; long versus normal r = 0.85, P<0.001; short versus long r = 0.85, P<0.001.

### Correlation analysis between angiogenic markers and miR-155

We have previously published on the prognostic impact of miR-155 in our large (335 patients) NSCLC population [37]. In this study we explored the correlation between miR-155 and several well-known angiogenic markers. The same cut-off values as previously published were used for FGF2 and miR-155 [33], [37]. Figure S1 shows *in situ* hybridization (ISH) analysis of NSCLC representing strong and weak intensities for tumor cell miR-155 expression as well as negative and positive controls. Table 5 shows the correlation between angiogenic markers [vascular endothelial growth factor - A (VEGF-A), platelet derived factor – B (PDGF-B), HIF-1α and fibroblast growth factor 2 (FGF2)] and miR-155. miR-155 correlated significantly with FGF2 in the total cohort (r = 0.17, P = 0.002), though most prominent in the subgroup with nodal metastasis (r = 0.34, P<0.001). The crosstabs in Table 6 and Table 7 show the correlation and distribution of high and low expression of FGF2 and miR-155.

**Table 5:** Correlation analyses between angiogenic markers and miR-155 expression in NSCLC patients.

	miR-155		
	Total cohort (n = 335)	N0 (n = 232)	N+ (n = 103)
**VEGF-A**	r = 0.09, P = 0.11	r = 0.05, P = 0.50	r = 0.18, P = 0.07
**PDGF-B**	r = 0.04, P = 0.49	r = 0.06, P = 0.37	r = 0.01, P = 0.96
**HIF-1α**	r = 0.02, P = 0.68	r = 0.02, P = 0.75	r = −0.1, P = 0.27
**FGF2**	**r = 0.17, P = 0.002**	r = 0.06, P = 0.35	**r = 0.34, P<0.001**

Abbreviations: VEGF-A, vascular endothelial growth factor-A; PDGF-B, platelet derived factor -B; HIF-1α, hypoxia inducible factor 1α; FGF2, fibroblast growth factor 2; N0, lymph node negative patients; N+, lymph node positive patients.

**Table 6:** Crosstab showing the correlation between miR-155 and FGF2 in the total cohort.

		miR-155		
		Low expression	High expression	Total
**FGF2**	Low expression	**156**	**137**	293
	High expression	**6**	**21**	27
	Total	162	158	320

Spearman correlation, r = 0.17, P = 0.002.

**Table 7:** Crosstab showing the correlation between miR-155 and FGF2 in patients with lymph node metastasis (N+).

		miR-155		
		Low expression	High expression	Total
**FGF2**	Low expression	**48**	**37**	85
	High expression	**1**	**13**	14
	Total	49	50	99

Spearman correlation, r = 0.34, P<0.001.

## Discussion

To our knowledge, this is the first NSCLC miR expression profiling study showing a gene set connected to angiogenesis-related miRs with the highest impact in pathway analysis. Consistent with previously published studies we observe significant differences in miR profiles between normal and tumor tissue and between samples from patients with a poor versus a favorable DSS. Several of these significant altered miRs are associated with angiogenesis and are interesting candidate markers for further evaluation. By validating one of these markers, miR-155, in a cohort of 335 NSCLC patients, we find this miR for the first time to be significantly associated with the well-known angiogenic marker FGF2.

The major weaknesses of the study in the search for novel candidate markers are the relatively heterogeneous study population and the low number of patients included in the analysis. Many of the results presented herein are therefore only borderline significant (or some only showing a trend) and further validation in a larger NSCLC set, as done for miR-155, is a prerequisite for firmer conclusions. This is especially the case when comparing significant differences between tumor tissues (long versus short survival) as there, in general, are larger differences between tumor tissues and normal controls. The fold changes are in average higher and the p-values stronger in Table 2 (tumor tissue versus normal controls) compared with Table 3 (long versus short survival). From a clinical point of view, at least when it comes to miRs as potential prognostic or predictive markers, miRs with altered expression between short versus long survival group are of special interest. We have therefore explored the literature on four angiogenesis related miRs from Table 3 (miR-106a, miR-155, miR-182 and miR-424) and validated miR-155 in a large set of NSCLC patients. In addition we wanted to highlight the potential impact of miR-21, miR-126 and miR-210 from Table 2 as there are convincing reports indicating these miRs to have potential impact in angiogenesis. Besides, we have previously shown miR-126 to have prognostic impact in our NSCLC cohort [38].

Strengthening our results are strong and significant correlations between the microarray hybridization data and the same miRs validated by qPCR. Further, the altered miR expressions in tumor versus normal tissue are to a large degree consistent with previously published NSCLC miR studies [15]–[17], [20] and as discussed in the following there are constantly new literature added, supporting our candidate miRs to be involved in angiogenesis.

Few studies have explored the impact of pathways related to miR gene targets in NSCLC. The interpretation of such analyses is not straight-forward as the results are highly dependent on which target genes are linked to the miRs and which miRs are connected to the different pathways. In addition miRs are often multifunctional and the same miR may target diverse genes in different pathways, and a gene may be targeted by several miRs. Although not statistically significant, the GSEA ranged angiogenesis as the most important pathway in our NSCLC samples. There are several novel reports connecting specific miRs to angiogenesis supporting our findings [23], [24], [39]–[42]. Besides, Raponi et al. have shown that the angiogenesis-related fibroblast growth factor (FGF) pathway had a significant gene enrichment in squamous NSCLC [17].

In a comprehensive murine study, Olson et al. identified specific miR expression signatures associated with steps in tumorigenesis and different hallmarks of cancer, including angiogenesis [23]. Angiogenesis–inhibiting signature miRs were evaluated by qPCR technique and defined as those miRs significantly altered in normal versus sunitinib-treated primary tumors by >1.3 fold change [23]. Sunitinib is a tyrosine kinase inhibitor targeting vascular endothelial growth factor receptor (VEGFR), platelet-derived growth factor (PDGFR) and c-Kit. In accordance with our qPCR validation results, PCR data in general often show an increased fold change compared with hybridization data. Many of the angiogenesis–related miRs observed by Olson and coworkers are also significantly altered in our material [23]. Among the most interesting angiogenesis-related miRs significantly altered in both studies we find miR-126, miR-155, and miR-21 and miR-424.

miR-126 has recently been reviewed as an important player in angiogenesis [43] and Wang et al. showed in a murine model that it enhances the proangiogenic actions of VEGF and FGF by repressing the expression of Spred-1, an intracellular inhibitor of angiogenic signaling [44]. miR-126 has also been located within the epidermal growth factor-like domain 7 (EGFL7) gene. EGFL7 may have a major role in angiogenesis by promoting VEGF signaling and vascular integrity [45]. Moreover, we have previously reported that miR-126 expression is significantly associated with VEGF-A in NSCLC [38]. Further, we observed that the prognostic impact of miR-126 is related to histological subtypes and nodal status. In addition the expression and roles of miR-126 may be different in various malignancies [23].

miR-155 is one of the miRs most consistently involved in neoplastic disease [46], but to our knowledge, not been related to angiogenesis prior to the study by Olson and coworkers [23]. It has been reported to be up-regulated in several human NSCLC studies [15], [17], [20], and down-regulated in a murine model when using the angiogenic inhibitor sunitinib [23]. In our array data, miR-155 tended to be significantly altered between the short and long survival groups (Table 3) and was significantly over-expressed in tumor versus normal tissue in the qPCR validation set. We have reported the prognostic impact of miR-155 to be associated with histological subtypes and nodal status but did not link these results to angiogenic markers [37]. Based on these new data we now examined whether miR-155 correlated to well-known angiogenic/hypoxic parameters as VEGF-A, PDGF-B, HIF-1α and FGF2. Interestingly, we observed, for the first time, miR-155 to be significantly associated with FGF2 with the highest impact in the N+ NSCLC subgroup. Though FGF2 has several functions, it is an important player in angiogenesis. In fact, miR-155 also tended to correlate with VEGF-A in the N+ subgroup. miR-155 was one of the first miRs to be associated with NSCLC development and further studies will be important in order to explore its potential angiogenic function.

In this study, miR-21 is significantly up-regulated in tumor versus normal tissue (Table 2), supporting previous NSCLC reports [15], [17], [20] and the observed down-regulation after anti-angiogenic treatment [23]. miR-21 has also been found to be highly expressed in endothelial cells [24]. In a recent study by Liu et al., miR-21 was overexpressed by transfecting pre-miR-21 into human prostate cancer cells and tumor angiogenesis was assayed using chicken chorioallantoic membrane [47]. They found that overexpression of miR-21 in DU145 cells increased the expression of HIF-1α and VEGF, and induced tumor angiogenesis. They conclude that miR-21 induces tumor angiogenesis through targeting PTEN, leading to activation of the AKT and ERK1/2 signaling pathways, and thereby enhancing HIF-1α and VEGF expression. The same pathways are described as important in NSCLC development as well [48], and further studies to address this relationship with miR-21 in lung cancer would be of interest.

The Olson study indicates miR-424 to be involved in angiogenesis as this miR was down-regulated after sunitinib treatment [23]. In addition, Ghosh et al. have studied this miR and described that hypoxia-induced miR-424 expression in human endothelial cells regulates HIF-α isoforms and promotes angiogenesis [49]. They further suggest miR-424 to play an important physiological role in post-ischemic angiogenesis. In contrast, Nakshima et al. reported down-regulation of miR-424 to contribute to the abnormal angiogenesis via MEK1 and cyclin E1 in senile hemangioma, demonstrating the potential tissue and stage specific impact of the same miR [50]. In our study, miR-424 was not altered when comparing tumor with normal tissue, but was significantly up-regulated in tissue from patients with poor prognosis compared to tissue from patients with a favorable prognosis (Table 3). There is to our knowledge no published study which has explored the prognostic impact of miR-424 in cancer. However, as there is a significant miR-424 up-regulation in the poor prognostic group it would be of interest to further evaluate this marker in a large scale NSCLC study.

Another significant up-regulated marker in our material is miR-210 (Table 2), which previously has been related to angiogenesis [41], [51], [52]. miR-210 up-regulation is believed to be a crucial element of endothelial cell response to hypoxia and therefore potentially important in tumor angiogenesis. This miR has recently been reviewed by Devlin et al. [51], concluding that miR-210 is a robust target of hypoxia-inducible factor and that its overexpression has been detected in a variety of cardiovascular diseases and solid tumors. In NSCLC cell lines, miR-210 is reported to mediate mitochondrial alterations associated with modulation of HIF-1 activity [52]. However, much of the functional and prognostic impact of miR-210 in NSCLC still remains unresolved.

miR-182 is included in the angiogenesis pathway in the GSEA, partly because fibroblast growth factor receptor substrate 2 (FRS2) is one of its target genes. FRS2 is a major regulator of the fibroblast growth factor pathway that we and others have shown to be important in NSCLC progression, potentially by stimulating angiogenesis [33], [53]. Interestingly, miR-182 was the only miR significantly altered in all three combinations as shown in the Venn diagram (Figure 1). Its up-regulation in tumor (both poor and favorable prognostic group, Table 2) when compared to normal tissue, is consistent with the previous study by Raponi et al. [17]. Moreover, miR-182 is significantly up-regulated in short DSS versus long DSS patients (Table 3). However, Barchack et al. observed higher miR-182 expression in primary tumors than in the metastatic lung tumors, indicating that miR-182 expression may reach a peak in early stage NSCLC.

We observe the same trend for miR-106a, which has been shown to be up-regulated during hypoxia in breast and colon cancer cell lines [40]. As previously reported, miR-106a was up-regulated in NSCLC [16], [17], [20]. We also observe its expression to be significantly augmented in tumors versus normal controls (Table 2). As for miR-182, miR-106a was further increased in tissue from patients with a short survival rather than long survival (Table 3).

As expected, many miRs known to be associated with angiogenesis were not significantly altered in our study. Cluster miR-17-92 (miR-17-5p, miR-17-3p, miR-18a, miR-19a, miR-19b, miR-20a and miR92-1), let-7, let-7f, miR-27b, miR-15b, miR-16, miR-92a, miR-99a, miR-130a, miR-214, miR-221, miR-222 and miR-296 as well as some of the miRs presented in the heat map (Figure 3), were not significantly altered or they exhibited only minor fold changes [24], [39], [41], [42]. This lack of significant alterations may be due to false negative results as there are few patients in each group. Furthermore, as many miRs appear to be tissue and stage specific, some of these miRs may alternatively exert an impact limited to subgroups of resected NSCLC or may be important in other malignancies.

Angiogenic inhibitors in combination with chemotherapy are established as NSCLC treatment, but a further knowledge on biology, effectors mechanisms and prognostic and predictive markers are warranted. miRs are stable and potentially measurable in both tissue and serum. Several miRs are linked to angiogenesis and this study supports the assumption that a number of angiogenesis-related miRs are potentially important in NSCLC progression. We propose miR-21, miR-106a, miR-126, miR-155, miR-182, miR-210 and miR-424 to be among the most interesting candidates. Since miRs often are stage and tissue specific, large scale studies are warranted to further explore their prognostic and predictive impact. Besides the same miR often has multiple target genes and different functions. Therefore functional studies are highly needed to gain better insight into their biological functions.

## Materials and Methods

### Ethics statement

The study is approved by The National Data Inspection Board, The Regional Committee for Research Ethics and Biobank Board Collection of Tissue. Information and subsequent written consent from patients or next of kin was considered, but as this was a retrospective study with more than half of patients deceased, some more than ten years ago, The Regional Committee for Research Ethics specifically waived the need for consent.

### Patients and tissue samples

From a previously well-described large NSCLC cohort [30], twenty formalin-fixed and paraffin-embedded (FFPE) tumor tissue samples were obtained from patients diagnosed with stage I-IIIA at the University Hospital of Northern Norway (UNN) and Nordland Central Hospital (NLCH). These were ten patients with a short disease-specific survival (DSS) (NSCLC_01 - NSCLC_10), and ten patients with long DSS (NSCLC_11 - NSCLC_20). In addition, samples from normal lung tissue sites in ten NSCLC patients (NSCLC_01, NSCLC_02, NSCLC_06, NSCLC_11, NSCLC_12, NSCLC_13, NSCLC_15, NSCLC_16, NSCLC_18 and NSCLC_19) were included in the study (NORM_01 - NORM_10). Four sample cores, each 0.6 mm in diameter, from viable tumor areas from each tumor and normal specimen were taken and pooled prior to RNA isolation in order to obtain a representative sample of each patient. Long and short DSS groups were stratified in order to get equal distribution of gender and histology. The tumor specimens were stored at the Departments of Pathology at UNN and NLCH, staged according to The International Union Against Cancer (UICC) TNM classification, and histologically subtyped according to the World Health Organization guidelines [54], [55]. All patient material was reviewed by two independent and trained pathologists (SAS and KAS). None of the patients received chemo- or radiotherapy prior to surgery. Complete patient data was obtained from medical records, including demographic, clinical, treatment and outcome data.

### RNA isolation

RNA was isolated from the collected core samples by using the Recover All™ Total Nucleic Acid Isolation Kit for FFPE Tissues (Ambion, Austin, TX), according to the manufacturer's instructions, with some minor adjustments. Xylene treatment was increased from 3 min to 8 min, and protease treatment was increased from 3 hrs to approximately 20 hrs. RNA quality and quantity was assessed by using the NanoDrop 1000 spectrophotometer (Thermo Fisher Scientific, Wilmington, DE) and further verified by an Agilent 2100 Bioanalyzer profile.

### Microarray procedures

Total RNA (700 ng) from sample and reference was labelled with Hy3™ and Hy5™ fluorescent label using the miRCURY^Tm^ LNA Array power labelling kit (Exiqon, Vedbaek, Denmark) following the procedure described by the manufacturer. The Hy3™-labeled samples and a Hy5™-labelled reference RNA sample (containing an equal aliquot of all RNA species included in this study) were mixed pair-wise and hybridized to the miCURY^Tm^ LNA Array version 5^th^ generation (Exiqon, Denmark), which contains capture probes targeting all human miRNAs registered in the miRBASE version 14.0 at the Sanger Institute. The hybridization was performed according to the miRCURY™ LNA array manual using a Tecan HS4800 hybridization station (Tecan, Austria). After hybridization the microarray slides were scanned and stored in an ozone free environment (ozone level below 2.0 ppb) in order to prevent potential bleaching of the fluorescent dyes. The miCURY™ LNA Array microarray slides were scanned using the Agilent G2565BA Microarray Scanner System (Agilent technologies Inc., USA) and the image analysis was carried out using the ImaGene 8.0 software (BioDiscovery Inc., USA). The quantified signals were background corrected with offset value 10 and normalized using the global Lowess (Locally Weighted Scatterplot Smoothing) regression algorithm [56].

### Database submission of microarray data

The microarray data were prepared according to minimum information about a microarray experiment (MIAME) recommendations [57] and deposited in the GEO database (http://www.ncbi.nlm.nih.gov/geo/). The GEO accession number for the platform is GSE27705 and the study may be viewed at: http://www.ncbi.nlm.nih.gov/geo/query/acc.cgi?acc=GSE27705.

### Quantification of mature miRNAs by real-time qPCR

One or several of the following criteria were used when selecting miRs for PCR validation; significant P-value, high fold change and/or angiogenesis-related miRs. Five samples from each group with sufficient tumor miR remaining after the array analysis were used in the PCR validation. Out of the 41 tested miRs, 28 miRs were detected in 7 or more samples and analysed. The stability of the miRNAs detected in all samples was tested by Exicon using the Normfinder software. miR-423-5p was one of the recommended reference miRNAs together with miR-150, miR-423-5p and miR-300. miR-300 was excluded due to very low levels detected. Further Normfinder found large variation in miR- 150, however miR-423-5p was found to be the most stable miRNA in the data set and consequently used as reference. All real-time qPCR experiments were performed by Exiqon, Vedbaek, Denmark. 10 ng RNA was reverse transcribed in 10 µl reactions using the miRCURY LNA™ Universal RT miR PCR, Polyadenylation and cDNA synthesis kit (Exiqon); each sample was processed in triplicates. cDNA was diluted 100× and 4 ul was used in 10 ul PCR reactions according to the protocol for miRCURY LNA™ Universal RT miR PCR; each miR was assayed once by qPCR in triplicate cDNA. The amplification was performed in a LightCycler® 480 Real-Time PCR System (Roche) in 384 well plates. LinRegPCR (version 11.5) software was used to determine the qPCR amplification efficiency. The average amplification efficiency was used to correct the Raw Cp values.

### Pathway analysis

The impacts of the different pathways were evaluated by Gene Set Enrichment Analysis (GSEA) derived from Protein ANalysis THrough Evolutionary Relationship (PANTHER). The PANTHER classification system is a unique resource that classifies genes by their functions, using published scientific experimental evidence and evolutionary relationships to predict function even in the absence of direct experimental evidence. The gene set was taken from the MirDB (http://www.mirdb.org) (http://rnajournal.cshlp.org/content/14/6/1012.full) database who has compiled gene lists from pathways curated in the PANTHER database.

A full list is available at http://mirdb.org/cgi-bin/pathway.cgi. miRNA annotation is based on miRBase version 13 (http://www.mirbase.org/). GSEA analysis was carried out using the R statistical language (www.r-project.org) and the GSEA package for R available at http://www.broadinstitute.org/gsea/downloads.jsp.

### Data analysis and statistical analysis

The data were analyzed using Linear Models and Empirical Bayes Methods for Assessing Differential Expression in Microarray experiments (LIMMA) [58], and significance was determined at the 0.1 level corrected for false discovery rate (FDR) using the method of Benjamini & Hochberg [59]. Principal component analysis (PCA) and Partial least squares (PLS) were carried out on the data in order to visualize the data structure and look for potential outlier samples [60].

The enrichment score (ES) reflects the degree to which a gene set is overrepresented at the top or bottom of a ranked list of genes. Normalized enrichment scores (NES) are used to compare analysis results across gene sets. GSEA accounts for differences in gene set size and in correlations between gene sets and the expression dataset [61]. The false discovery rate (FDR) is the estimated probability that a gene set with a given NES represents a false positive finding. The nominal p- value estimates the statistical significance of the enrichment score for a single gene set.

Pearson's correlation coefficient, r, was used to describe the association between array data and results from qPCR. Pearson Chi-Square and Fisher Exact Test were used to correlate angiogenic markers with miR-155.

## Supporting Information

Figure S1 ***In situ*** **hybridization (ISH) analysis of NSCLC representing strong and weak intensities for tumor cell miR-155 expression.** Negative (scramble-miR) and positive (U6) controls from the same tissue area are shown. Strong miR-155 staining (A) with corresponding negative (C) and positive (E) controls to the left. Weak miR-155 staining (B) with corresponding negative (D) and positive (F) controls to the right. ISH positive signals (miR-155 and U6) stain blue, while nuclei stain red.
(TIF)

Table S1 **The 128 significant -up and down-regulated (−1, 1) miRs are presented with FDR adjusted p-values and respective fold changes (log2 scale).**
(XLS)

Table S2 **Comparison between the array and the qPCR data in validation set.** The differences in average expression levels between the sample groups in the array study are shown in columns 2–4 (dLMR values = log2 scale). The differences in average expression levels between the sample groups in the qPCR study are shown in columns 5–7 (ddCp values = log2 scale). Positive numbers >1 are shown in red ( = fold change >2), negative numbers <−1 ( = fold change >−2).
(DOC)
