# Supplementary figures and images for: MicroRNA Signatures in Tumor Tissue Related to Angiogenesis in Non-Small Cell Lung Cancer
Source: PLoS One. 2012 Jan 25;7(1):e29671. doi: 10.1371/journal.pone.0029671 (PMC3266266; doi:10.1371/journal.pone.0029671)

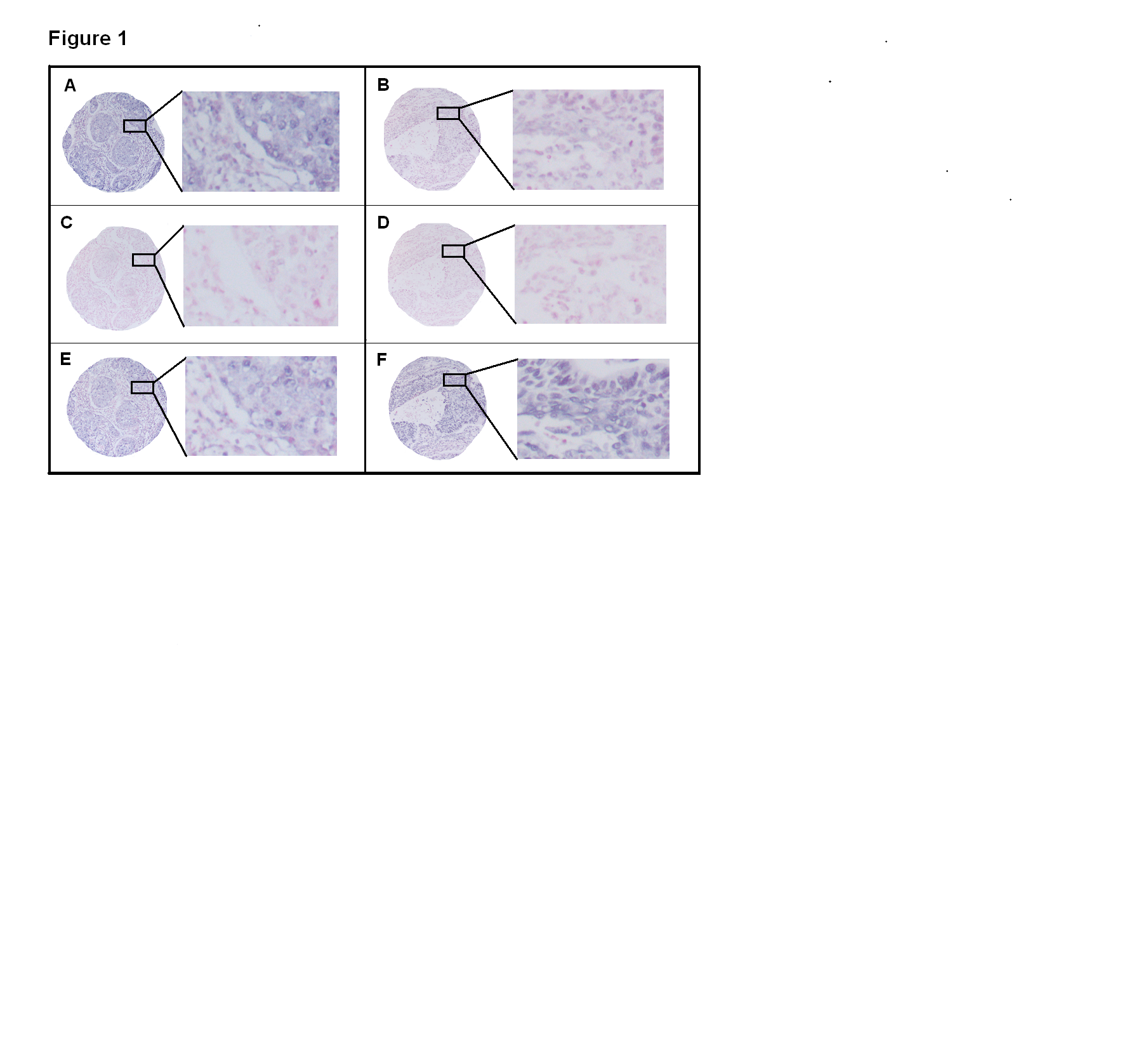

Supplement: Figure S1 — In situ hybridization (ISH) analysis of NSCLC representing strong and weak intensities for tumor cell miR-155 expression. Negative (scramble-miR) and positive (U6) controls from the same tissue area are shown. Strong miR-155 staining (A) with corresponding negative (C) and positive (E) controls to the left. Weak miR-155 staining (B) with corresponding negative (D) and positive (F) controls to the right. ISH positive signals (miR-155 and U6) stain blue, while nuclei stain red. (TIF) [file pone.0029671.s001.tif]
