# Supplementary material for: MicroRNA Signatures in Tumor Tissue Related to Angiogenesis in Non-Small Cell Lung Cancer
Source: PLoS One. 2012 Jan 25;7(1):e29671. doi: 10.1371/journal.pone.0029671 (PMC3266266; doi:10.1371/journal.pone.0029671)
Supplement: Table S2 — Comparison between the array and the qPCR data in validation set. The differences in average expression levels between the sample groups in the array study are shown in columns 2–4 (dLMR values = log2 scale). The differences in average expression levels between the sample groups in the qPCR study are shown in columns 5–7 (ddCp values = log2 scale). Positive numbers >1 are shown in red ( = fold change >2), negative numbers <−1 ( = fold change >−2). (DOC) [file pone.0029671.s003.doc]

**Supplementary Table 2**

|  | **Array - dLMR** | | | **qPCR – ddCP** | | |
| --- | --- | --- | --- | --- | --- | --- |
| miR | Long vs normal | Short vs normal | Short versus long | Long vs normal | Short vs normal | Short vs long |
| **150** | -0.047 | -0.110 | -0.062 | 0.155 | -3.089 | -3.244 |
| **145** | -0.901 | -1.009 | -0.107 | -1.859 | -1.862 | -0.002 |
| **106a** | 0.489 | 1.211 | 0.72 | 0.750 | 2.835 | 2.085 |
| **93** | 1.091 | 1.164 | 0.073 | 1.493 | 2.096 | 0.604 |
| **210** | 0.484 | 0.676 | 0.192 | 2.211 | 2.436 | 0.225 |
| **126*** | -0.704 | -0.947 | -0.243 | -1.804 | -1.058 | 0.747 |
| **142-3p** | 0.290 | -0.698 | -0.989 | 0.807 | -1.183 | -1.990 |
| **99b** | -0.430 | -0.032 | 0.398 | -0.498 | 0.559 | 1.057 |
| **126** | -1.447 | -1.668 | -0.222 | -1.454 | -1.487 | -0.033 |
| **193b** | 0.407 | 1.105 | 0.698 | 0.913 | 2.170 | 1.257 |
| **365** | -0.073 | 0.346 | 0.420 | -0.469 | 1.656 | 2.125 |
| **143** | -0.799 | -1.061 | -0.261 | -1.072 | -1.004 | 0.068 |
| **574-3p** | -0.312 | 0.036 | 0.348 | -1.461 | 0.583 | 2.044 |
| **151-5p** | -0.021 | 0.596 | 0.617 | -0.338 | 1.101 | 1.439 |
| **125a-5** | -0.874 | -0.717 | 0.157 | -0.806 | -0.200 | 0.605 |
| **205** | 2.342 | 1.073 | -1.269 | 4.345 | 1.880 | -2.466 |
| **451** | -1.376 | -1.984 | -0.608 | -1.823 | -1.693 | 0.131 |
| **Let-7a** | -0.691 | -0.174 | 0.517 | -0.717 | 1.087 | 1.804 |
| **21** | 1.634 | 1.742 | 0.108 | 1.516 | 1.637 | 0.122 |
| **155** | 0.140 | -0.206 | -0.346 | 1.143 | 0.118 | -1.024 |
| **424** | -0.144 | 0.404 | 0.548 | 0.463 | 1.300 | 0.837 |
| **29c** | -0.373 | -0.927 | -0.553 | -0.771 | -1.072 | -0.300 |
| **378** | 0.372 | -0.119 | -0.491 | 0.478 | -0.401 | -0.879 |
| **720** | 0.541 | 0.028 | -0.513 | 0.277 | 0.294 | 0.017 |
| **182** | 0.731 | 1.677 | 0.946 | -0.637 | 1.081 | 1.718 |
| **31** | 0.442 | 1.481 | 1.039 | -0.818 | 3.099 | 3.918 |
| **Let-7f-1*** | 0.098 | -0.169 | -0.268 | -0.838 | 0.395 | 1.233 |
| **30a** | -1.149 | -1.232 | -0.083 | -1.281 | 0.131 | 1.412 |

Comparison between the array and the qPCR data in validation set. The differences in average expression levels between the sample groups in the array study are shown in columns 2-4 (dLMR values - log2 scale). The differences in average expression levels between the sample groups in the qPCR study are shown in columns 5-7(ddCp values = log2 scale). Positive numbers > 1 are shown in red (= fold change > 2), negative numbers > 1 are shown in blue (= fold change > 2).
